# A comparison of infectivity between polyhedra of the *Spodoptera litura* multiple nucleopolyhedrovirus before and after passage through the gut of the stink bug, *Eocanthecona furcellata*

**DOI:** 10.1093/jis/14.1.96

**Published:** 2014-07-18

**Authors:** R. K. Gupta, Mudasir Gani, P. Jasrotia, K. Srivastava, V. Kaul

**Affiliations:** 1 1Division of Entomology, Sher-e-Kashmir University of Agricultural Sciences and Technology-Jammu, Chatha, Jammu and Kashmir, India – 180009; 2 2GLBRC Research Coordinator, W.K. Kellogg Biological Station; Michigan State University; 3700 E Gull Lake Dr Hickory Corners, MI 49060

**Keywords:** SpltMNPV before passage, SpltMNPV after passage, LD _50_, ST _50_

## Abstract

Infectivity of polyhedra of *Spodoptera litura* multiple nucleopolyhedrovirus before and after passage through the gut of the predatory stink bug, *Eocanthecona furcellata* Wolff (Hemiptera: Pentatomidae) was compared through field bioassay studies. Three sets of *E. furcellata* were used for bioassays and these were allowed to feed on a single meal of five third instar Oriental leaf worm, *Spodoptera litura* (Fabricius) (Lepidoptera: Noctuidae), that were infected with polyhedra before passage, after passage, and healthy (control) larvae 1 day prior to the trial. The predators were subsequently released on cabbage plants that were infested with 100 healthy *S. litura* larvae. The median lethal dose (LD _50_ ) and survival time (ST _50_ ) values before and after passage through the gut were not significantly different. Additional mortality due to virus infection increased 13– 17% before and after treatments but within these treatments the mortality did not vary significantly. It was concluded that *E. furcellata* disseminated the virus through their feces into the ecosystem and infectivity of the SpltMNPV was not altered after passage through the gut of the predator.

**Abbreviations: NPV** , nucleopolyhedrovirus; **SpltMNPV** , *Spodoptera litura* multiple nucleopolyhedrovirus; **PIBs** , polyhedral inclusion

## Introduction

Baculoviruses are a group of double-stranded DNA viruses with a narrow host range. They are well known due to their potential as agents of biological control of pests in agriculture and forestry. The predatory bug *Eocanthecona furcellata* Wolff (Hemiptera: Pentatomidae) is regarded as potential predator of pest insects at both the nymphal and adult stages. *E. furcellata* is a generalist predator; it may consume any soft-bodied arthropods it encounters in the ecosystem.

Predator-mediated baculovirus dispersal has been demonstrated to be an important mechanism for virus dissemination in diverse crop and forest habitats ( Entwistle et al. 1993 ; Fuxa and Richter 1994 ). This dispersal occurs through the excretion of viable viral polyhedra in their feces for periods of several days following ingestion of baculovirus-infected meal ( Beekman 1980 ; Young and Yearian 1987 ; Vasconcelos et al. 1996 ). Natural enemies may become superficially contaminated during the consumption of infected prey, and may physically disperse the virus over plant surfaces ( Sait et al. 1996 ).

Although the consumption of infected larvae and subsequent release of viable virus by predatory bugs are likely to occur in nature, the ecological implication of this phenomenon is poorly understood. The importance of predaceous arthropods in the dissemination of insect viruses depends on three factors: the acceptance of virus-infected insects as food, the effect of passage through the predator gut on virus infectivity, and the interactive behavior of predator and prey in relation to virus acquisition. However, effective integration of baculoviruses into existing pest management systems depends on their compatibility with other components of the systems. A full understanding of the spread of baculovirus by predators is therefore necessary for the successful application of biopesticides and also to predict the environmental fate of natural and genetically modified microorganisms ( Fuxa and Ritcher 1994 ). For instance, some parasitoids and predators could be affected detrimentally by the pathogens infecting their prey ( Cossentine and Lewis 1988 ; Ruberson et al. 1991 ); few reports suggested that they were not affected by the pathogens ( Young and Hamm 1985 ; Sajap 1989 ). Insect predators that feed on virus-infected larvae can acquire moderate to high levels of infection ( Young and Yearian 1987 , 1992), and the release of viable virus in the predator feces occurs for at least four days after feeding on infected larvae ( Beekman 1980 ). It is not known, however, whether infectivity of the virus is altered after passage through the gut of insect predators. In this study, we used a bioassay method to observe the infectivity of *Spodoptera litura* multiple nucleopolyhedrovirus (SpltMNPV) before and after passage through the gut of *E. furcellata* and investigated the role of predator-mediated virus dissemination in enhancing pest suppression in the field.

## Materials and Methods

### Host insect *(Spodoptera litura)*

The *Spodoptera litura* (Fabricius) (Lepidoptera: Noctuidae) insects used in this study were from the offspring of 34 ^th^ generation female moths obtained from the Indian Agriculture Research Institute, New Delhi. These insects had been continuously maintained at 28 ± 2°C and 60% RH with a 16:8 L:D photoperiod on artificial diet as described by Shorey and Hale (1965) with certain modifications ( Gupta et al. 2010 ). Egg masses were collected and surface-sterilized with 0.01% sodium hypochlorite. The larvae were reared in a plastic container (30 × 20 × 10 cm) lined with a paper towel at the bottom. The diet and the paper were changed on alternate days.

### Predator *(Eocanthecona furcellata)*

The predator used in this study was from the offspring of 20 ^th^ generation female bugs that emerged from field-collected eggs laid on *Parthenium* leaves ( Gupta et al. 2004 ). The predators were maintained in the laboratory at 28 ± 2°C and 60% RH with a 16:8 L:D photoperiod and fed every 24 hours on healthy 10-day-old *S. litura* larvae.

### The insect virus

The SpltMNPV used in this study was originally isolated from naturally-infected (in the field) *S. litura* larvae collected from tomato fields in Jammu, India ( Monobrullah et al. 2007**)** . The virus was propagated in *S. litura* larvae maintained on a semi-synthetic diet of chickpea flour. Viral polyhedra were extracted by homogenizing virus-killed larvae in 0.1% sodium dodecyl sulphate (SDS), followed by filtration through muslin cloth and subsequent pelleting through continuous sucrose (40 to 66% w/w) gradient centrifugation for 1 hr at 50,000g. After several washes in TE (10 mm Tris-HCl, pH 8, 115 1 mm EDTA), the polyhedra were resuspended in a small volume of distilled water and stored in aliquots at -20°C. The virus was quantified using a haemocytometer and phase contrast microscope at ×800 magnification.

### Infectivity of polyhedra of SpltMNPV before and after passage through the gut of *E. furcellata*

To observe the infectivity of SpltMNPV before and after passage through the gut of *E. furcellata,* two sets of SpltMNPV were used in the study: one set consisting of SpltMNPV before passage and the other after passage through the gut of *E. furcellata* . To obtain the SpltMNPV after passage, *E. furcellata* was allowed to feed on moribund infected fourth-instar *S. litura* and placed in individual petri dishes (80 mm × 15 mm). Feces were collected up to 48 hours after feeding on infected prey, as no significant loss of infectivity of polyhedra occurred during passage through the gut within 24–48 hours. For feces collection, predators were used only once throughout the experiment. Feces were titrated in 100 µL of sterile water and each suspension was assayed for viable virus by feeding the virus to each of fifty second instar host larvae using the diet plug system ( Gupta et al. 2013 ). Circular discs measuring 1.5 cm in diameter were cut from the diet and contaminated with serial concentrations of SpltMNPV. For both cases, five doses of SpltMNPV ranging from 3,000 to 187.5 polyhedral inclusion bodies (PIBs) per larva were used. 15 µL of each viral concentration were pipetted onto each diet disc. These discs were air-dried and then placed individually in a compartmentalized 2 × 2 cm acrylic insect rearing system (NBAII, www.nbaii.res.in ). One larva was then placed in each compartment. A total of 100 larvae were used for each concentration. Larvae, having eaten the entire diet disc within 24 hours, were transferred to fresh uncontaminated diet. Mortality was recorded daily until pupation. Dead larvae were checked for SpltMNPV infection. The experiment was replicated five times. A preliminary experiment comparing the estimation of polyhedra in a series of dilutions by the above method and by haemocytometer count indicated a highly significant regression coefficient ( *P* < 0.001).

### Field infectivity of polyhedra of SpltMNPV before and after passage through the gut of *E. furcellata*

To compare the ability of *E. furcellata* to disperse the virus, three sets of predators that were fed with polyhedra before passage, after passage through the gut, and healthy hosts (control) were released in the field on individual cabbage plants that did not show signs of *S. litura* infestation. The plants were randomly selected and allocated to three different treatments. Each experimental cabbage plant was at an average distance of 170 cm (or seven cabbage plants) away from any other experimental plant, such that each plant represented a single independent replicate. One day prior to the trial, pre-starved adult predators (three days old) were fed a single meal of five healthy or SpltMNPV-infected (before passage and after passage) third instars of *S. litura* (48 hr postinfection) of uniform size and equal weight (65-70 mg). The infected larvae that were offered to predators were treated with a similar dose of 1 × 10 ^3^ PIBs/insect using the diet plug system as described above. Only those predators that consumed all the larvae within 24 hours were used in the experiment. Treatments involved placing one adult and 100 healthy third instar *S. litura* from the laboratory culture on the top third portion of the cabbage plant, which was then enclosed by a nylon mesh bag 35 cm tall and 30 cm wide, gently tied at the base to minimize insect movement. Each treatment was replicated five times. After six days, the bags were opened, the predators were removed, and remaining *S. litura* larvae were recovered, taken to the laboratory, and individually transferred to semi-synthetic diet and checked daily for virus-induced mortality for 10 days. All virus deaths were confirmed by microscopic examination through Giemsa staining.

### Data analysis

The data were subjected to Probit analysis (SPSS-16) for the calculation of median lethal dose (LD _50_ ± 95% CL) and median survival times (ST _50_ ); corresponding confidence interval for each dose was calculated using log rank test under Kaplan-Meier analysis. Data on cumulative mortality percentage of *S. litura* larvae caused by SpltMNPV before and after passage through the gut of *E. furcellata* were subjected to a “pairwise” *t* -test. The data on recovery and viral mortality of *S. litura* in the field were subjected to one-way ANOVA.

## Results

The effect of virus dose on the cumulative mortality was significant both for before passage (F _(4,__20)_ = 50.823, *P* = 0.001) and after passage (F _(4,__20)_ = 56.6684, *P* = 0.001) of SpltMNPV through the gut of *E. furcellata* . A gradual increase in the cumulative mortality was observed as the dose increased from 187.5 to 3,000 PIBs/larva in both cases, with a significant positive correlation (r values: 0.906 before passage and 0.836 after passage) between dose and mortality. However, when the cumulative mortality percentage of *S. litura* larvae caused by SpltMNPV before and after passage through the gut of *E. furcellata* was compared among similar doses, no significant difference was observed ( Table 1 ). Probit analysis of the mortality data showed median lethal dose (LD _50_ ) estimates of 817 and 1,144 PIBs/larva before and after passage of SpltMNPV through the gut of the predator, with confidence limits of 532–1107 and 904– 1418 respectively, but as the confidence limits overlapped, the differences in infectivity were not significant.

**Table 1.**
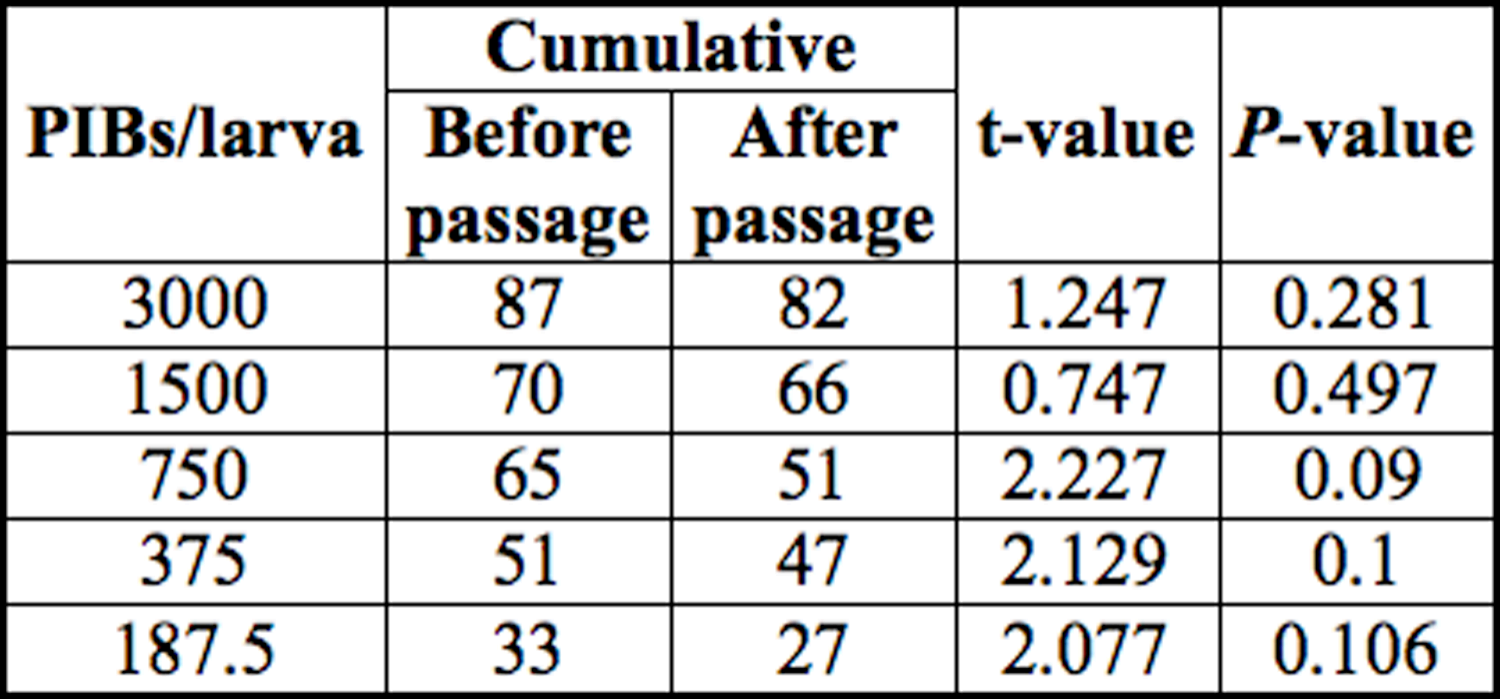
Cumulative mortality (%) of *Spodoptera litura* larvae caused by SpltMNPV before and after passage through the gut of *Eocanthecona furcellata.*

It was also found that higher doses led to faster mortality. The ST _50_ values for the respective doses (3000, 1500, 750, 375, and 187.5 PIBs/larva) were 155.4, 163.6, 173.8, 201.4, and 209.6 hours before passage, respectively, and 162.6, 171.2, 177.0, 214.4, and 231.2 hours, respectively, after passage of SpltMNPV through the gut of *E. furcellata* ( Table 2 ).

**Table 2.**
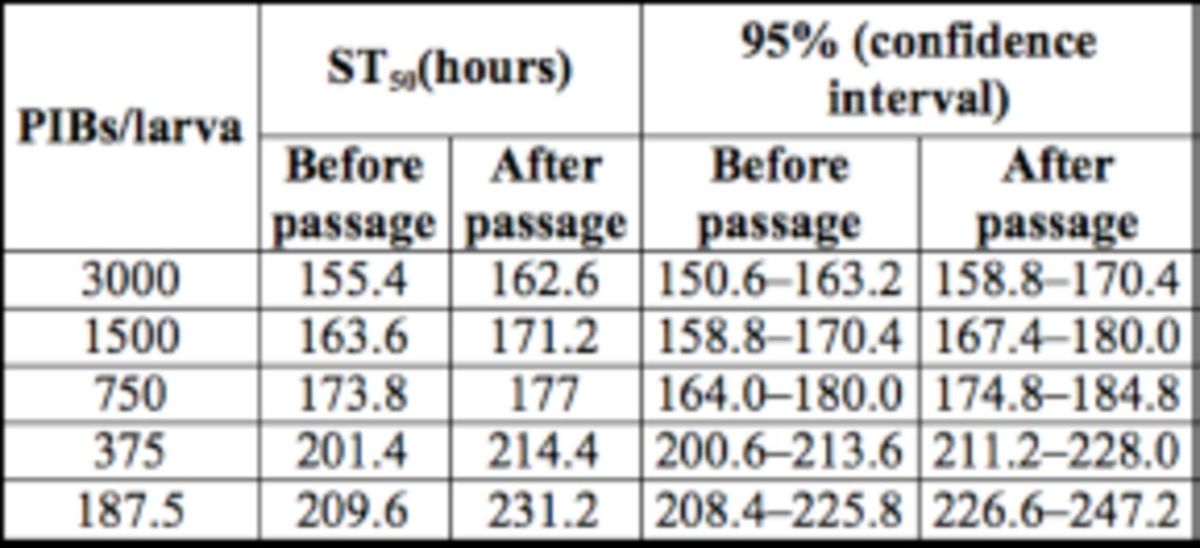
Estimates of the median survival time (ST _50_ ) of SpltMNPV before and after passage through the gut of *Eocanthecona furcellata* and 95% confidence interval as obtained from log rank test under Kaplan-Meier analysis *.*

In the field trial, the percent infection due to SpltMNPV in recovered larvae was significantly higher (13–17%) on plants where virus-fed predators were released when compared to plants that hosted healthy predators that did not show viral infection in recovered larvae (F _(2,__12)_ = 4.12, *P* = 0.001). However, when the viral mortality was compared on plants that hosted predators that were fed with two kinds of polyhedra (the original PIBs that were not passed through the gut and the PIBs that were subsequently passed through the gut of *E. furcellata* ), the observed differences were not significant ( Table 3 ).

**Table 3.**
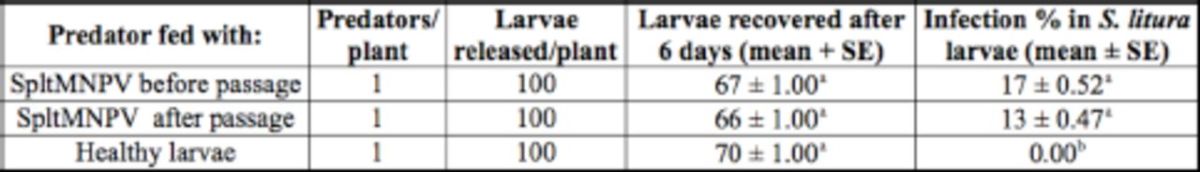
Infection in *Spodoptera litura* larvae (%) by polyhedra in feces of *Eocanthecona furcellata* adults fed with SpltMNPV before passage, SpltMNPV after passage, and healthy *S. litura* in the field.

*Means in the same column followed by the same letters are not significantly different from each other (ANOVA; Tukey’s HSD; *P* > 0.05), SE = standard error of means.

## Discussion

The results suggest that PIBs of SpltMNPV were still infectious and the infectivity of the virus was not altered after passage through the gut of the predator. The observed values for LD _50_ of SpltMNPV before and after passage through the gut of an insect predator did not differ significantly. Further, at similar virus doses, ST _50_ values of SpltMNPV before and after passage through the gut of the insect predator were not significantly different from each other. It is well established that after feeding on infected larvae, predators excrete viable baculovirus in their feces ( Boucias et al. 1987 ; Young and Yearian 1987 ; Fuxa et al. 1993 ; Vasconcelos et al. 1996). These predators apparently do not suffer significant deleterious effects from consuming virus-infected lepidopteran larvae ( Heinz et al. 1995 ; McNitt et al. 1995 ; Li et al. 1999 ). This is likely because the gut of most insect predators is acidic, in contrast to the highly alkaline guts of phytophagous lepidoptera that degrade the proteinaceous matrix of the viral polyhedra, resulting in the release of virions and the subsequent infection of host midgut cells ( Granados and Lawler 1981 ). However, Castillejos et al *.* (2001) suggested that infectivity was not related solely to gut pH, as the activity of a virus following passage through the gut was maintained in *Doru taeniatum* , but not in *Chrysoperla rufilabris* , for reasons that are not clear as both predators have acidic midguts. Similarly, Vasconcellos et al *.* (1996) showed that *Harpalus rufipes* could excrete viable virus for up to 15 days after feeding on an infected prey. However, Cooper (1981) suggested that infectivity of the virus is reduced during prolonged retention in the alimentary tract of the predator, presumably as a result of enzymatic activity. These studies established that there is a loss of infectivity over time, but did not focus on the infectivity of virus before and after passage through the gut of the predator. A loss of infectivity was attributed to a reduction of the number of polyhedra in feces over time following ingestion of baculovirus-infected meal.

These findings were further substantiated through field trials that demonstrated an additional mortality due to virus infection (up to 17%) on those plants where the predators were fed with the two kinds of polyhedra (the original PIBs that were not passed through the gut and the PIBs that were subsequently passed through the gut of *E. furcellata).* However, when the viral mortality was compared among these two sets of predators, the observed differences were nonsignificant. As no significant variation in larval recovery up to six days was observed, we assume that there was nearly equal predation by virus-fed and non-virus-fed bugs. Mortality in the field, although not high, showed that predators released infective virus in a form that could be acquired by susceptible lepidopteran hosts. Previous experiments involving the release of virus-fed predators (Carabidae) into the field that were subsequently infested with host larvae resulted in lower levels of infection (< 5%) of larvae (Vasconcelos et al. 1996; Castijellos et al. 2001). This is because in comparison to carabids, pentatomids are quite mobile, exhibiting frequent contact between predator and prey with a mean searching time of 3.5 minutes. This higher infectivity was due to higher mobility of the predator as compared to host larvae, which may increase the speed of inoculum dispersal (Vasconcellos et al *.* 1996). In field conditions, virus disseminated in the feces of insect predators is sufficient to initiate epizootics in larval populations. This has been reported in predators such as *Sycanus leucomesus* Walk. ( Sajap et al *.* 1999 ), *Calosoma sycophanta* (L.) ( Capinera and Barbosa 1975 ), *Podisus maculiventris* Say ( Abbas and Boucias 1984 ), and *Nabis roseipennis* (Reuter) ( Young and Yearian 1987 ). In our previous studies, we found that the survival of predators that fed on infected hosts was not affected by the ingestion of NPV. The present study suggests that *E. furcellata* could also serve as a carrier and disseminator of the virus through its feces without any loss of infectivity.

## Abbreviations

NPV: nucleopolyhedrovirus
SpltMNPV: Spodoptera *litura* multiple nucleopolyhedrovirus
PIBs: polyhedral inclusion bodies
